# Virus-induced congenital malformations in cattle

**DOI:** 10.1186/s13028-015-0145-8

**Published:** 2015-09-24

**Authors:** Jørgen S. Agerholm, Marion Hewicker-Trautwein, Klaas Peperkamp, Peter A. Windsor

**Affiliations:** Section for Veterinary Reproduction and Obstetrics, Department of Large Animal Sciences, Faculty of Health and Medical Sciences, University of Copenhagen, Dyrlaegevej 68, 1870 Frederiksberg C, Denmark; Department of Pathology, University of Veterinary Medicine Hannover, Bünteweg 17, 30559 Hannover, Germany; Department of Pathology, GD Animal Health, Arnsbergstraat 7, P.O. Box 9, 7400 AA Deventer, The Netherlands; Faculty of Veterinary Science, University of Sydney, Camden, NSW 2570 Australia

**Keywords:** Bovine, Teratogen, Schmallenberg virus, Blue tongue virus, Bovine virus diarrhea virus, Akabane virus, Aino virus, Hydranencephaly, Hydrocephalus, Cerebellar hypoplasia, Porencephaly, Arthrogryposis

## Abstract

Diagnosing the cause of bovine congenital malformations (BCMs) is challenging for bovine veterinary practitioners and laboratory diagnosticians as many known as well as a large number of not-yet reported syndromes exist. Foetal infection with certain viruses, including bovine virus diarrhea virus (BVDV), Schmallenberg virus (SBV), blue tongue virus (BTV), Akabane virus (AKAV), or Aino virus (AV), is associated with a range of congenital malformations. It is tempting for veterinary practitioners to diagnose such infections based only on the morphology of the defective offspring. However, diagnosing a virus as a cause of BCMs usually requires laboratory examination and even in such cases, interpretation of findings may be challenging due to lack of experience regarding genetic defects causing similar lesions, even in cases where virus or congenital antibodies are present. Intrauterine infection of the foetus during the susceptible periods of development, i.e. around gestation days 60–180, by BVDV, SBV, BTV, AKAV and AV may cause malformations in the central nervous system, especially in the brain. Brain lesions typically consist of hydranencephaly, porencephaly, hydrocephalus and cerebellar hypoplasia, which in case of SBV, AKAV and AV infections may be associated by malformation of the axial and appendicular skeleton, e.g. arthrogryposis multiplex congenita. Doming of the calvarium is present in some, but not all, cases. None of these lesions are pathognomonic so diagnosing a viral cause based on gross lesions is uncertain. Several genetic defects share morphology with virus induced congenital malformations, so expert advice should be sought when BCMs are encountered.

**Electronic supplementary material:**

The online version of this article (doi:10.1186/s13028-015-0145-8) contains supplementary material, which is available to authorized users.

## Background

Bovine congenital malformations (BCMs) usually occur sporadically although some herds may experience an increased number of cases over time. Farmers may in such situations seek veterinary advice regarding the cause of the malformation. Veterinarians may also be faced with the challenges of BCMs during their examination of cattle, e.g. in association with dystocia. A pathomorphological diagnosis can usually be established in cases of central nervous system (CNS) and musculoskeletal malformations as such disorders are often readily recognisable. However, farmers and their veterinarians are usually much more interested in the aetiological diagnosis, which is generally more difficult to establish under field conditions.

The diagnosis of BCMs, especially virus-induced congenital malformations (VICMs), may be achieved by submitting appropriate materials to diagnostic laboratories, although this is rarely done systematically due mainly to the costs of shipment of specimens and laboratory analyses. It is tempting for the veterinary practitioner to offer an aetiological diagnosis based only on the clinical findings and gross lesions. However, the diagnosis of BCMs is a specialised field where significant knowledge of genetic and teratogenic syndromes may be required to achieve an accurate diagnosis. Detailed knowledge on BCMs is generally not a key competency of veterinarians in large animal clinical practice as they only face these disorders sporadically. Suggesting an aetiological diagnosis from limited observations and insufficient knowledge on the causes and morphology of the wide spectrum of bovine congenital syndromes, may lead to misdiagnosis.

A particular challenge for veterinarians and laboratory diagnosticians is the differentiation of “true” VICMs (i.e. where a viral causal association can be established) from BCMs where exposure to a potential teratogenic virus during foetal development has occurred but the infection did not cause the pathology observed. Precolostral calves having VICM may either have congenital antibodies against a teratogenic virus or be virus-positive, as determined by viral nucleic acid detection [polymerase chain reaction (PCR)] or culture. Being antibody- or virus-positive does not necessarily imply that the virus caused the malformation as the outcome of a foetal infection with a teratogenic virus depends on factors such as the foetal age at exposure and the teratogenic properties of the virus strain in particular [1–4]. Some calves having a congenital defect may be antibody- or virus-positive simply by coincidence, i.e. reflecting the prevalence of intrauterine exposure in the general population and having been infected outside the gestational “window” of viral teratogenic effects. Interpretation of findings in BCM cases having consumed colostrum is complicated as antibodies may be of maternal origin and because antibodies may mask the presence of virus. Furthermore, some calves may have both VICM and BCM of another cause, creating more complications in achieving a diagnosis. It is tempting to associate the findings of antibodies or virus with the malformation, but unless there is scientific evidence for such an association, such as pathology consistent with viral lesions, this should be done with caution. Knowledge on the morphology of VICM in calves is crucial when interpreting the significance of antibodies or virus in cases of BCM.

This paper aims to provide an overview of the basic knowledge required by bovine practitioners and laboratory diagnosticians on the gross morphology of VICM in cattle. This is supported by general information on the viruses, pathogenesis, etc. to achieve a better understanding. Diagnostic challenges are presented together with details on known genetic diseases of cattle sharing major features with VICMs. The most important teratogenic viruses of bovines, especially in a European context, are reviewed to assist readers to recognise and investigate VICMs in cattle, ensuring these disorders are correctly differentiated from BCMs due to other causes.

## Search strategy

This critical review is based on a search in PubMed (http://www.ncbi.nlm.nih.gov/pubmed) using the terms “cattle, bovine, malformation, congenital” combined with viral names: Schmallenberg virus, blue tongue virus, bovine virus diarrhea virus, Akabane virus, and Aino virus. The title and abstract of the obtained hits were evaluated and articles referring to the gross morphology were obtained and assessed in detail. In addition, our own archives were used as a source of additional information. Our extensive experience with BCMs and VICMs were used to critically evaluate the literature and our personal photo archives were used to illustrate the characteristics of VICMs in cattle.

## Review

Bovine foetal infection with bovine virus diarrhea virus (BVDV), Schmallenberg virus (SBV), blue tongue virus (BTV), Akabane virus (AKAV), or Aino virus (AV), is associated with a range of congenital malformations of which the most prominent develop in the CNS, especially in the brain, with accompanying lesions frequently developing in the musculoskeletal system. A brief overview of the lesions is presented in Table 1, while an in-depth description is provided in the text and figures.

**Table 1:** Definition of teratogenic lesions in the central nervous, muscle and skeletal systems and their association with intrauterine infection with bovine virus diarrhea virus (BVDV), Schmallenberg virus (SBV), blue tongue virus (BTV), Akabane virus (AKAV), or Aino virus (AV)

Lesion	Definition	BVDV	SBV	BTV	AKAV/AV
Hydranencephaly	Extensive loss of cerebral tissue with replacement by clear fluid	×	×	×	×
Porencephaly	Cystic fluid filled cavities in the brain tissue	×	×	×	×
Hydrocephalus	Dilation of the lateral ventricles by cerebrospinal fluid	×	×	×	
Microencephaly	Reduced size of the cerebrum	×	×	×	×
Cerebellar hypoplasia	Reduced size of the cerebellum	×	×	×	
Kyphosis	Dorsal vertebral column curvature		×		
Lordosis	Ventral vertebral column curvature		×		
Scoliosis	Lateral vertebral column curvature		×		
Torticollis	Twisted cervical vertebral column curvature		×		
Arthrogryposis	Joint contraction of the limbs		×		×

### Bovine virus diarrhea virus

Bovine virus diarrhea virus belongs to the genus *Pestivirus* of the family *Flaviviridae*. Based on antigenic and genetic characteristics, two genotypes (species), BVDV-1 and BVDV-2, have been identified. Within each genotype, two biotypes characterized by their ability to damage cultured cells have been found, i.e. cytopathogenic (cp) and non-cytopathogenic (ncp) biotypes [5].

The most commonly reported BVDV genotype is BVDV-1 and, although BVDV-2 strains in general are thought to be more virulent, both BVDV genotypes can induce the same spectrum of disease manifestations [3]. BVDV genotypes 1 and 2 and their two biotypes occur worldwide [6], although the infection has been almost eradicated in some countries [7].

The pathogenesis of BVDV infection in cattle is complex and infections with the ncp biotype belonging to either BVDV-1 or -2 genotypes occurring before and during gestation may result in a wide range of clinical and pathological presentations [7]. The outcome of infections of pregnant cattle depends on the immunity of the dam, stage of gestation, the immunocompetence of the foetus, the virus biotype, and the virulence of the strain [2]. Transplacental infection of the developing foetus with ncp BVDV during the first 6 months of gestation may result in embryonic death, mummification, abortion, persistent infection, premature birth of a live but weak or undersized or apparently normal appearing calf, and various congenital malformations [2, 3, 6, 8, 9]. Teratogenic effects in the bovine foetus after infection of the dam with ncp BVDV have been reported to occur between gestation days (GDs) 79 and 150 and most commonly affect the CNS, which at that time is still in the process of growth and differentiation [2, 3]. The pathogenetic mechanisms of congenital defects are thought to result from a combination of direct cellular damage and inflammatory responses of the foetus to the virus [2]. The virus destroys immature foetal neuronal and neuroglial cells, causes failure of migration of these cells, and induces destruction of brain parenchyma. In the cerebrum, loss of brain tissue and failure of development leads to the formation of cavitating lesions, i.e. hydranencephaly and porencephaly [10]. BVDV-induced necrosis of external granular layer cells in the cerebellum and failure of these cells to migrate results in depletion of granule cells within the developing internal granular layer. Also, ectopia and degenerative changes of Purkinje cells occur. Inflammatory vascular lesions leading to folial oedema, haemorrhages, and ischaemia are responsible for destruction of cerebellar folia and cavitating lesions in the cerebellar parenchyma [10]. The outcome of these lesions is a cerebellum of reduced size grossly recognized as cerebellar hypoplasia. Further congenital malformations after infection of bovine foetuses with BVDV include ocular lesions and alterations of thymus, bones, hair coat, lung, and kidneys [2].

**Fig. 1:**
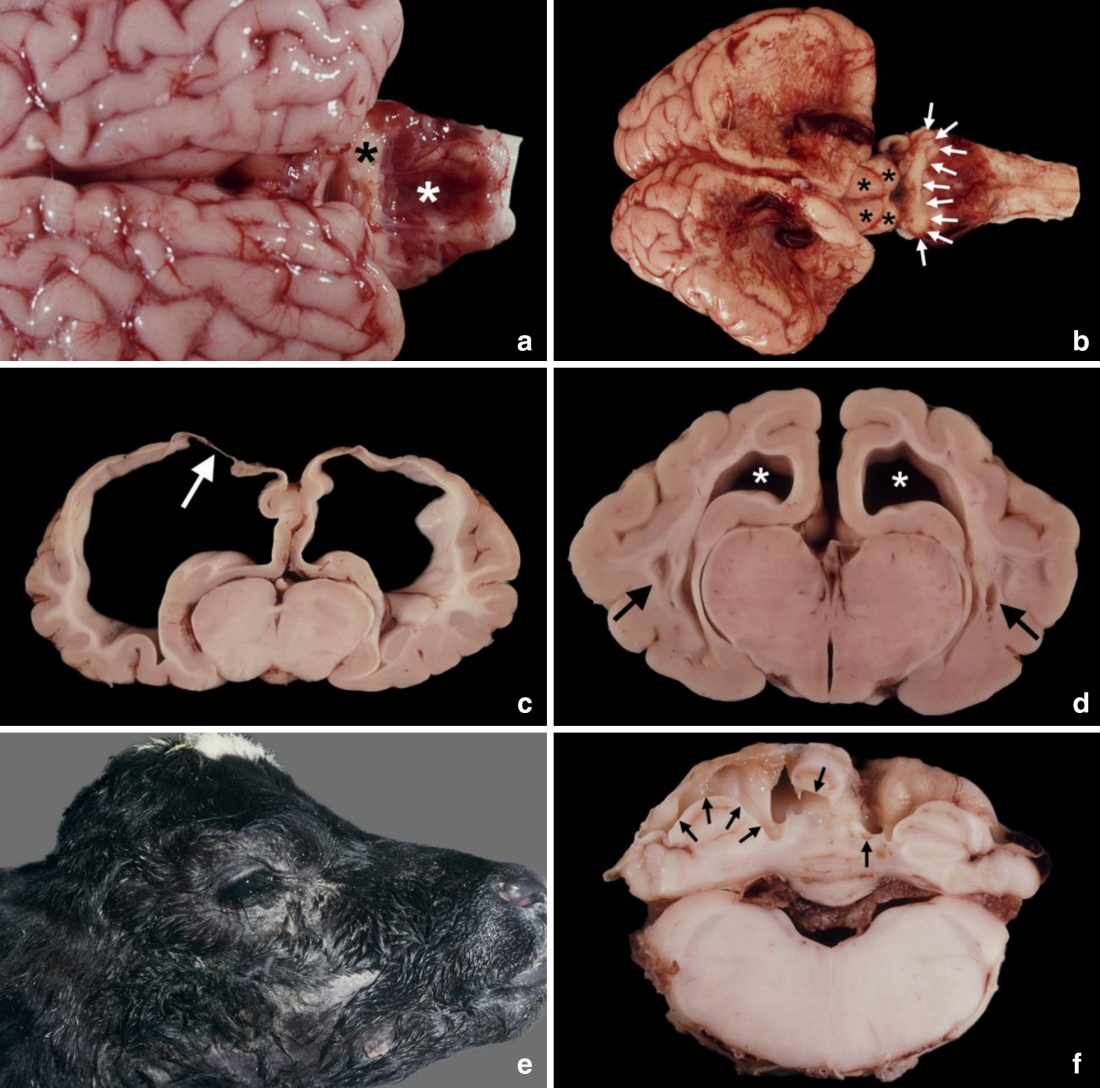
Teratogenic lesions associated with intrauterine infection with bovine virus diarrhea virus (BVDV) in cattle. The lesions are representative also for other teratogenic viruses infecting cattle. **a** Severe cerebellar hypoplasia. There are only small irregular parts of the developing cerebellum (*black asterisk*). Posterior to the cerebellar tissue, the 4th ventricle is visible which is covered by congested choroid plexus tissue (*white asterisk*). **b** Hydranencephaly. The posterior and dorsal parts of the cerebral hemispheres are collapsed, partially ruptured and covered by thin membranes consisting of congested leptomeningeal tissue. The cerebellum is severely hypoplastic and reduced to a small, narrow mass of tissue (*arrows*). Between the posterior parts of the collapsed cerebral hemispheres and the hypoplastic cerebellum the rostral and caudal colliculi (*asterisks*) of the quadrigeminal plate are visible. **c** Hydranencephaly. There is marked bilateral thinning of the grey and white matters of the cerebral brain tissue surrounding the extremely dilated lateral ventricles. The dorsal parts of the cerebral hemispheres are partially covered by thin, partly translucent leptomeninges. Beneath the meninges the brain tissue is focally absent (*arrow*). Transverse brain section at the level of the optic chiasm. **d** Bilateral internal hydrocephalus. There is slight dilation of both lateral ventricles (*asterisks*). In the cerebral white matter bilateral porencephalic cysts are present (*arrows*). **e** Doming of the calvarium due to hydrocephalus. **f** Porencephalic cysts in the cerebellum. Cut surfaces of cerebellar tissue reveal the presence of variable-sized cystic cavities affecting the cerebellar hemispheres and the vermis (*arrows*). **a**–**e** Spontaneous foetal infection with BVDV, **f** Experimental foetal BVDV infection

The most frequent brain malformation after foetal spontaneous or experimental infection with BVDV is cerebellar hypoplasia [11–21]. Macroscopically, a considerable variation in the extent of hypoplastic cerebellar lesions can be found. In severe cases, only small nubs of cerebellar tissue are present, in which regular differentiation into lobes or folia is absent. In such brains, the choroid plexus is visible on the base of the 4th ventricle (Fig. 1a). In less severe cases of cerebellar hypoplasia, larger parts of the vermis and of the hemispheres are present. In other cases the malformed cerebellum shows only slight, uniform or irregular reduction in size of individual lobes and folia. Most cases of BVDV-induced cerebellar hypoplasia are associated with alterations of the cerebrum, i.e. hydranencephaly, hydrocephalus, or microencephaly [21–23].

In hydranencephaly, the cerebral hemispheres are transformed into large sac-like fluid-filled cavities, which are covered by thin, partly translucent leptomeninges (Fig. 1b, c). In such cases, upon severing the head from the vertebral column and also during opening the skull, clear, watery cerebrospinal fluid drain off from the vertebral canal and from the sac-like cavities. The cerebral lesions most severely affect the posterior and dorsal parts of the hemispheres (Fig. 1b). Transverse sections of hydranencephalic brains reveal severely dilated lateral ventricles and presence of remnants of atrophic cerebral brain tissue (Fig. 1c).

In cases of hydrocephalus, the lateral ventricles are slightly or markedly dilated with accumulation of clear cerebrospinal fluid (Fig. 1d). Macroscopically, depending on the degree of ventricular enlargement, the hydrocephalus can be associated with the presence of an enlarged and dome-shaped calvarium (Fig. 1e).

Microencephaly, characterized by subnormally-sized cerebral hemispheres occurs less frequently [21, 23]. Porencephaly is characterized by the presence of circumscribed variable-sized cystic cavities, which may occur in the cerebral hemispheres or in the cerebellum (Fig. 1d, f). Porencephalic cysts in brains of BVDV infected calves are located in the white matter alone or both in the grey matter (cortex) and white matter and, depending on their size, are macroscopically visible or can be found at microscopical examination [21, 23]. In brains with hydrocephalus, cavitating lesions may also be present in the periventricular brain parenchyma (porencephaly) (Fig. 1d) [21]. This suggests that the dilation of the ventricles represents hydrocephalus *ex vacuo*, i.e. the dilation of the ventricles is due loss of periventricular parenchyma. Both in spontaneous and experimental cases of bovine BVDV infection, hypomyelination of brain and spinal cord white matter has been described [24–28]. In case of hypomyelination, macroscopic abnormalities are absent and detection requires histological examination and confirmation of deficiency of stainable myelin by special staining (e.g. Luxol fast blue stain) of CNS tissue.

Congenital ocular lesions after spontaneous or experimental BVDV infection of bovine foetuses are commonly associated with cerebellar hypoplasia known as oculo-cerebellar syndrome [13, 14, 18, 19]. Macroscopically, bilateral microphthalmia and/or cataracts may occur, and histologically retinal dysplasia, optic neuritis, and atrophy can be found [2]. Other, congenital malformations that have been associated with BVDV infection include thymic hypoplasia, brachygnathia inferior, changes of the hair coat (hypotrichosis/alopecia, curly hair), hypoplasia of lung, renal dysplasia, deranged osteogenesis (growth arrest lines), and growth retardation [2, 29]. These lesions can occur either as single entities or can be associated with CNS or ocular lesions.

### Schmallenberg virus

Schmallenberg virus is a member of the genus *Orthobunyavirus*, characterized by three genomic ssRNA segments, and has been classified within the species Sathuperi virus of the Simbu serogroup [30]. SBV was discovered in 2011 as a novel and emerging pathogen in ruminants in north-western Europe and appeared as the first orthobunyavirus on this continent [31].

Several orthobunyaviruses of the Simbu serogroup show teratogenic properties in ruminants and transplacental orthobunyaviral infections, resulting in congenital malformations, have been reported in Asia, Australia, Africa and the Middle East [32–34].

Like most orthobunyaviruses, SBV is transmitted by *Culicoides* spp. biting midges [35–37] and a rapid spread of SBV in vectors and hosts occurred in Europe, following its first detection in Germany and The Netherlands [38–40]. Although the origin of SBV has not been elucidated, the virus was introduced in the region of the German-Dutch borders [41]. Within 2 years, SBV disseminated into 27 European countries, indicating a very efficient transmission through the arthropod vector [35, 41].

Hosts are infected during the vector active period and naïve adult cattle show none or only mild clinical signs such as transient fever, diarrhea, anorexia and reduced milk production during 3–11 days [41–43]. Infection during the gestation period may lead to transplacental infection of the foetus, however, the rate of vertical transmission seems low [44]. Teratogenic effects depend on the foetal developmental stage at the time of infection and as neuronal cells in the developing CNS are the target cells [45], infection results in a syndrome of congenital hydranencephaly and arthrogryposis. Furthermore, the foetal bovine immune system, being developed between approximately GDs 40 and 175, is capable to react with a CNS inflammatory response [46]. CNS lesions develop after infection between GDs 60 and 180, the vulnerable period of the foetal CNS [47, 48]. The severity of lesions in the brain and spinal cord depends on a complex interaction between foetal neurogenesis and immunocompetency and virulence of the strain [46]. SBV infection during early gestational stages results in severe dysplastic CNS lesions, whereas late gestational infections lead to encephalomyelitis [49].

Like most viral infections, transplacental transmission of SBV does not elicit placentitis and most malformed calves are stillborn at term. The weight of malformed calves is significantly less than normal with a correlation between the body mass deficit, the severity of the malformations and the amount of skeletal muscles [50].

**Fig. 2:**
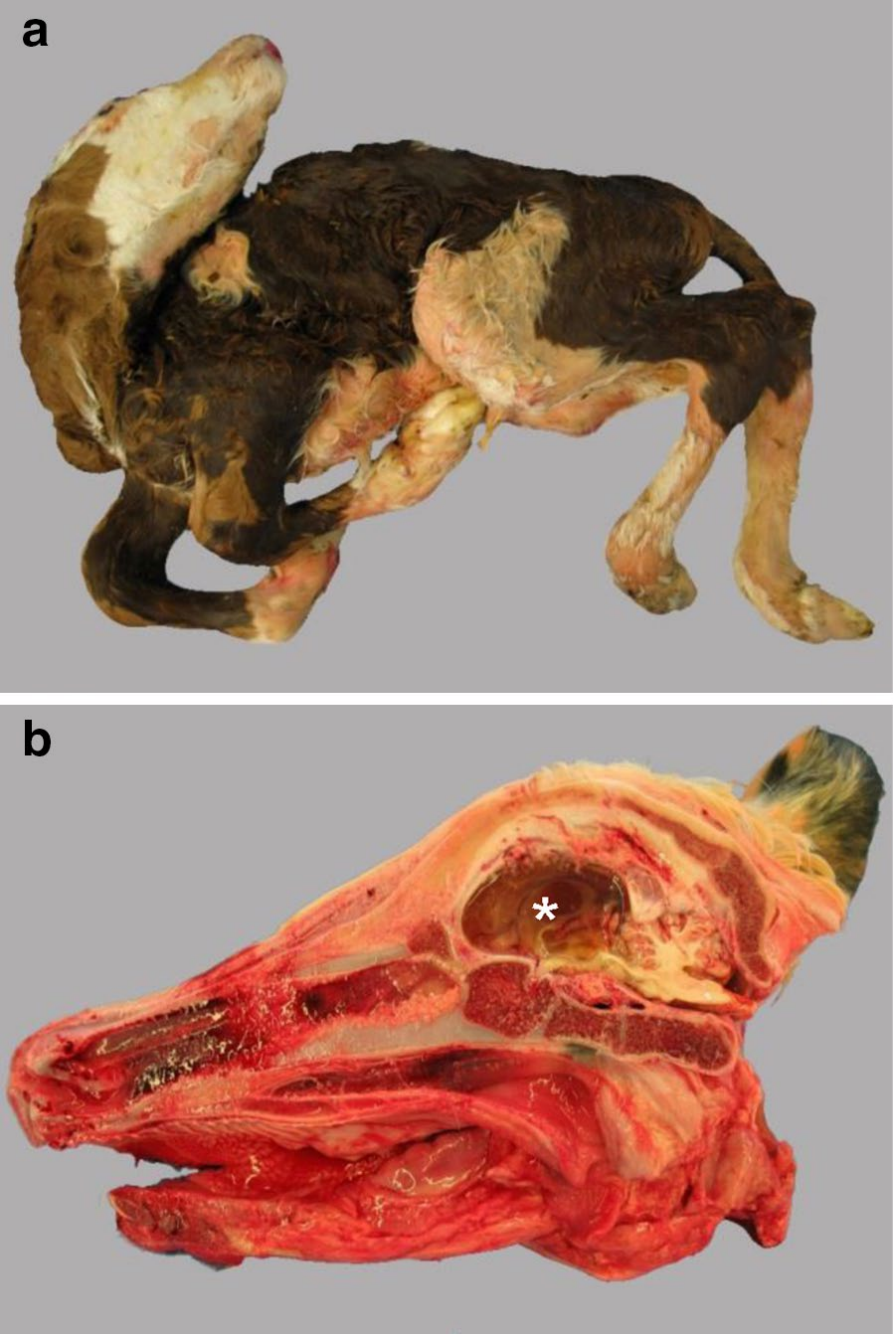
Teratogenic lesions associated with intrauterine infection with Schmallenberg virus (SBV) in cattle. **a** Generalised arthrogryposis of the appendicular skeleton (arthrogryposis multiplex congenita) and vertebral column malformation, including torticollis and kyphoscoliosis. **b** Hydranencephaly, brachygnathia inferior and thickened flat bones of calvarium. Parasagittal section through the head exposing the severely dilated right lateral ventricle (*asterisk*). **a**–**b** Spontaneous foetal infection with SBV

Malformations of the vertebral column and arthrogryposis, which are regarded secondary to dysplastic CNS lesions, are the most conspicuous exterior gross lesions in affected calves (Fig. 2a). The most frequently observed malformation of the vertebral column is torticollis, often in combination with scoliosis and/or kyphosis of the thoracic part of the vertebral column. Thoracic vertebral column malformations are often associated with a flattened ribcage. Scoliosis, kyphoscoliosis and kyphosis without torticollis appear less frequently and lordosis of the thoracolumbar part is observed sporadically. Congenital arthrogryposis of all four limbs (arthrogryposis multiplex congenita) appears in various degrees with bilaterally symmetric arthrogryposis in both fore- and hindlimbs as the most frequently observed malformation of the extremities. Sometimes, arthrogryposis is only present in both forelimbs or, rarely, only in both hindlimbs. Occasionally, unilateral arthrogryposis occurs, again mainly, if present, in the forelimbs. In most malformed calves congenital arthrogryposis is accompanied with vertebral column malformations, whereas vertebral column malformations without arthrogryposis are only observed sporadically [49, 50].

The axial and appendicular bones are normally founded and the vertebral malformations and arthrogryposis develop due to imbalance in foetal muscular activity (flexor vs. extensor muscles) occurring secondary to loss of neurons. In the brain and spinal cord, neuron loss is reflected in both the loss of descending tracts in the ventral spinal cord white matter and in the loss of ventral horn motor neurons in the spinal cord grey matter and in deficient ventral spinal nerve roots. Loss of spinal cord tissue, grossly visible in severely affected cases as a small dorsoventral flattened spinal cord (micromyelia), accounts for failure of normal skeletal muscle development due to denervation of the axial and appendicular musculature and hence to vertebral column malformation and arthrogryposis, respectively. Skeletal muscle tissue of arthrogrypotic limbs is hypoplastic, reflecting the extent of spinal cord dysplasia in the cervical and lumbar intumescences [49, 50].

Occasionally, a flattened skull and brachygnathia inferior are found. Deformation of the skull is assessed in combination with brain malformations. Neuroparenchymal loss in the brain may be visible by more or less severe defects in the grey and white matter of the cerebrum, cerebellum, midbrain, brainstem and medulla. Cerebral defects range from microencephaly, porencephaly in combination hydrocephalus and lack of gyri (lissencephaly) to hydranencephaly with only a small rim of cortical tissue left (Fig. 2b). Cerebellar hypoplasia is mainly observed in combination with cerebral defects, which also accounts for dysplasia of the midbrain, brainstem and medulla [49, 50].

### Blue tongue virus

Bluetongue virus, the causative agent of the disease bluetongue (BT) in ruminants, is a member of the genus *Orbivirus* within the *Reoviridae* family, with a genome, consisting of 10 dsRNA segments. Currently, 26 serotypes are distinguished, determined by variations of the outer-capsid protein VP2, which induces specific virus-neutralising antibodies [51]. The global distribution of BTV in temperate and (sub)tropical zones coincides with the presence of *Culicoides* spp. biting midges, transmitting BTV between ruminant hosts. The regional presence of the various BTV serotypes is associated with particular *Culicoides* spp. [52]. BT most commonly occurs in sheep, whereas cattle, acting as reservoir, sporadically develop clinical signs. The clinical outcome of a BTV infection varies with virulence and transmission potential of the infecting strain (phenotype) and depends on host (species, breed, age, immune status), vector and environmental factors [4, 53]. After infection of a susceptible host, BTV disseminates via the regional lymphnode to various organs, especially the lung and spleen. Subsequently, BTV replicates in mononuclear leukocytes and endothelial cells, organizing vascular injury, coagulopathy and hypovolemic shock [52].

Seasonal outbreaks of BT due to BTV serotypes 1, 2, 4, 9 and 16 have appeared since 1998 in the Mediterranean Basin at the boundaries of the temperate zone [52]. However, in 2006 BTV serotype 8 (BTV-8) emerged unexpectedly in north-western Europe, far beyond the previously known geographic area of BT and being an unknown serotype within Europe [54–56]. Initially, the morbidity and mortality rate in sheep and cattle appeared moderate, compared with other BTV-serotypes, but the severity and impact increased in the following 2 years [57, 58]. Clinical signs, also obvious in cattle, included fever, salivation, facial oedema, lesions on lips and nostrils, ulcerations in the oral and nasal mucosa, including tongue and gingiva, and coronitis [54]. Yet, in addition, BTV-8 exhibited an ability to cross the ruminant placenta, an unusual and unprecedented property of wild-type BTV, causing congenital brain malformations [59–65].

Experimental infections with various BTV-serotypes, using direct inoculation in bovine foetuses, have demonstrated that infection early in gestation, i.e. approximately between GDs 70 and 130, results in brain malformations. Infection during this time window causes hydranencephaly, occasionally combined with cerebellar defects, or leads to porencephaly and hydrocephalus [1]. As immunocompetency develops, foetal infection during the second half of gestation, i.e. from GD 145 onwards, may also be followed by clearance of the virus, resulting in a seropositive but PCR-negative live-born calf. However, acute infection at the final stage of gestation could lead to a seronegative PCR-positive offspring [63]. A seronegative PCR-positive calf could also indicate the existence of immunotolerance and hence persistently infected calves, though to date no convincing evidence has been presented for this phenomenon [56].

In cattle transplacental transmission with high rates ranging from 16 % [66] to 35 % [61, 67] not only causes reduced fertility and abortion but also results in a significant higher risk of a PCR-positive newborn calf, when infected in the second half of gestation compared to infection in the first half of gestation. Follow-up of 37 seemingly healthy PCR-positive calves up to 5 months after birth showed that BTV-8 was not detectable any longer by PCR at the end of the sampling period [68]. Apparently, seemingly healthy PCR-positive calves may be the outcome of foetal infection during the second half of gestation whereas foetuses with cerebral defects, infected during the vulnerable time window of CNS development at the first half of gestation, are also able to survive an intrauterine BTV-8 infection. These latter calves may show behavioural abnormalities, including dullness, inability to stand and suck well, disorientation and impaired vision, also referred to as “dummy calf”. Pathological changes in these cases, up to many months after birth, are confined to hydranencephaly, reflecting the predominant involvement of the cerebral cortex in interpretation of stimuli and behavioural patterns, and the function of intact remains of the brain, performing vital functions and locomotion [69].

Teratogenic BTV affects neuronal and glial precursor cells in the brain. Furthermore, vascular injury and infarction in the cerebrum may contribute to cerebral defects [69, 70]. Foetuses infected at later stages of gestation show encephalitis but no brain malformations [70–72].

Lesions in aborted or stillborn bovine foetuses associated with spontaneous foetal BTV-8 infections mainly consist of hydranencephaly, while porencephaly is observed less frequent. This probably reflects the foetal gestational age at the time of infection as seen in experimental infections [1]. Both lesions may be accompanied by cerebellar hypoplasia, which vary in severity from mild to almost complete lack of cerebellum. A slightly domed calvarium is present in some cases of hydranencephaly [59].

**Fig. 3:**
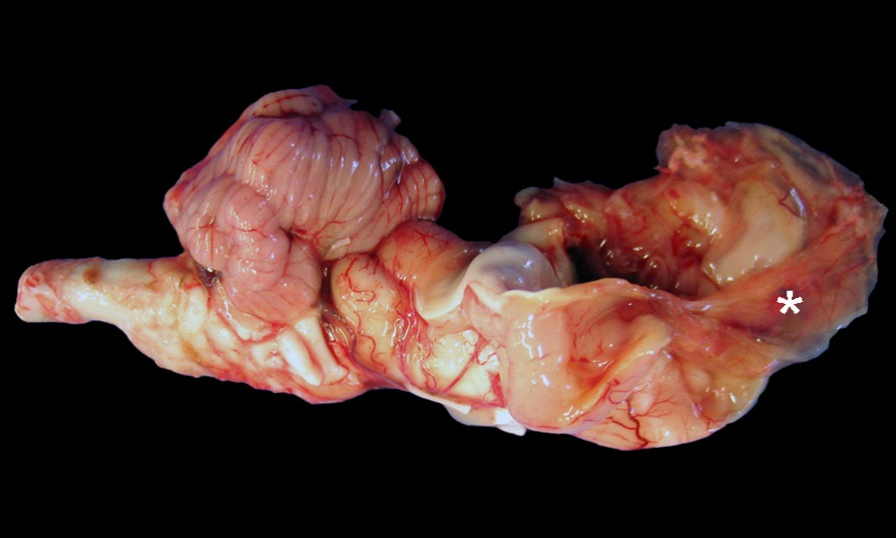
Hydranencephaly associated with intrauterine infection with bluetongue virus (BTV) in cattle. Specimen of the right half of the brain of a 4-days-old calf, born 2 weeks before term with nervous symptoms and blindness. The dorsal part of the cerebral hemisphere is ruptured and collapsed (*asterisk*) while the midbrain, brainstem, cerebellum and medulla oblongata are not affected. Spontaneous foetal infection with BTV

In liveborn intrauterine BTV-8 infected calves, surviving for up to several months, the dominating lesion is hydranencephaly (Fig. 3), although some cases may have hydrocephalus, probably due to periventricular loss of tissue [59, 60, 69].

Intrauterine BTV-8 infections do not cause significant lesions to the brainstem, medulla and spinal cord and hence are not associated with musculoskeletal disorders such as arthrogryposis and vertebral column malformations.

### Akabane and Aino viruses

The arthropod-borne Akabane virus (AKAV) and Aino virus (AV) are members of the genus *Orthobunyavirus*, and are the most pathogenic members of the Simbu serogroup in the family *Bunyaviridae*. Their principal vectors are *Culicoides* spp. biting midges, particularly *Culicoides brevitarsis*. AKAV was first suspected of causing congenital abnormalities in 1974 in Japan, with the distinct syndromes of hydranencephaly and arthrogryposis, sometimes accompanied by other abnormalities including abortion, stillbirth, polioencephalomyelitis and possibly microencephaly, frequently observed in parts of Australia [73–75]. AV is related to AKAV and has been occasionally associated with hydranencephaly and arthrogryposis in Australia but is antigenetically and biologically distinct [76], as is the closely related Chuzan virus [77].

**Fig. 4:**
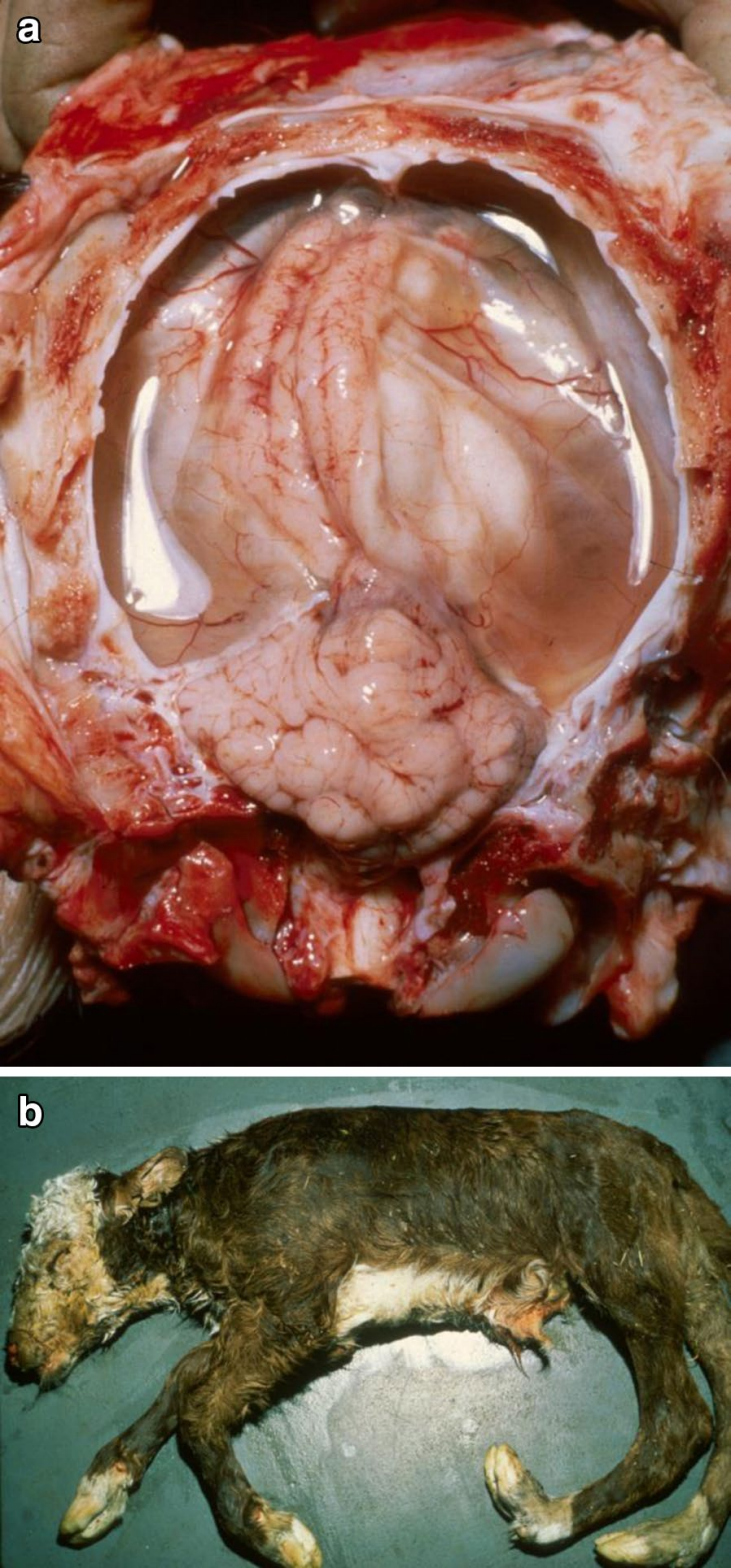
Teratogenic lesions associated with intrauterine infection with Akabane virus (AKAV). **a** Exposure of the brain after removal of the dorsal aspect of the skull. The hemispheres are partly collapsed due to severe hydranencephaly. Fluid is oozing out the brain cavities (due to damage to the vulnerable remnants of the cerebrum during opening of the skull) and surrounds the brain. Notice the normal sized cerebellum. Hydranencephaly usually develops in foetuses infected with AKAV between days 79 and 104 in gestation. **b** Generalised arthrogryposis of the appendicular skeleton, which typically develops in foetuses infected with AKAV between days 103 and 174 in gestation

AKAV is widely distributed in tropical and subtropical regions of Australia where it regularly infects young female cattle prior to gestation. Thus clinical signs are rarely observed in these endemic zones above the so-called “brevitarsis line” (see Additional file 1) with the exception of introduced pregnant naïve cattle. However, AKAV is one of the most potent teratogens of domestic ruminants and has been isolated from cattle, sheep and goats, with infection of naïve pregnant cattle associated with an occasional severe epizootic, or more regular sporadic outbreaks of hydranencephaly or arthrogryposis (Fig. 4a, b), with the nature of the lesions dependent on the stage of gestation when the cattle were infected. AKAV is also suspected of causing abortion, premature births, stillbirths and possibly other congenital abnormalities of the CNS, including porencephaly and microencephaly [78].

An investigation of a bovine epizootic of congenital hydranencephaly and arthrogryposis in New South Wales (NSW), Australia resulted in the identification of five groups of foetal abnormalities, corresponding to the gestational age of infection, from late (group 1) to early (group 5) [73]. Group 1 lesions comprised a microscopic non-suppurative encephalomyelitis; group 2 lesions were characterised by loss of spinal cord ventral horn motor neurons and Wallerian-type degeneration of ventral spinal nerves, resulting in ataxia, flaccid paralysis or mild arthrogryposis; group 3 was similar to group 2, but more severe; group 4 featured arthrogryposis, hydranencephaly and sometimes porencephaly while group 5 was similar to group 4, but more severe. Subsequent observations suggest that hydrocephalus with doming of the skull is rare in AKAV infection and when observed, involves severe dilation of the lateral ventricles that is presumably secondary to blockage of CSF flow in cases of severe hydranencephaly (PAW, unpublished observations). Further, malformations of the vertebral column are infrequently observed in AKAV infection, with spinal lesions mostly limited to microscopic changes including depletion of ventral horn neurones, associated with arthrogryposis [74].

A prospective study in the Hunter Valley coastal region of NSW in eastern subtropical Australia, enabled an understanding of the sequence of lesions induced in the bovine foetus under field conditions [74]. In this region, *C. brevitarsis* is suspected of over-wintering in protected valleys, with peak activity in the summer period causing infection with AKAV usually before or during the mating period so that heifers are immune at or shortly after joining. However, outbreaks of hydranencephaly and arthrogryposis occur when naïve pregnant animals are exposed to the virus, either by “spill-over” of the insect vectors from the endemic geographic areas of the coast to further inland where naïve cattle reside, or when naïve pregnant animals are transported from a non-vector area into AKAV endemic areas for agistment and other purposes, particularly during drought [75, 79]. Changes in the distribution of the vector occur from year to year and concern that changing climatic conditions can result in the sudden exposure of large numbers of naïve animals to viral infection and an epizootic outbreak of disease occur, ensures that both arthropods populations and serological responses to the arboviruses are monitored regularly throughout Australia by the National Arbovirus Monitoring Program (NAMP).

In summary, AKAV outbreaks have been recorded commonly in coastal NSW in eastern Australia and occasionally beyond in eastern Australia as a result of movement of pregnant cattle into the endemic region or movement of insect vectors beyond the AKAV endemic region. Seroconversion and disease are strongly linked to the presence of *C. brevitarsis* [80].

As with other teratogenic viruses, the pathogenic outcome of infection depends on the age of foetal exposure. In cattle, infection between GDs 79 and 104 results in hydranencephaly, whereas exposure at GDs 103-174 causes arthrogryposis, with focal Wallerian-type degeneration in the brain and spinal cord [74]. Clinical signs of AKAV infection are not usually observed during the postnatal viraemic period in cattle in Australia, although encephalomyelitis has been suspected and is occasionally seen in calves [81] and adult cattle [82] infected post-natally in Japan.

### Congenital syndromes not proven as VICM

Calves with congenital malformation syndromes of non-viral cause may be exposed to a potential teratogenic virus during foetal development by accident. These calves are thus born with evidence of congenital infection, confirmed by either demonstration of congenital antibodies by serology or virus infection by culture or PCR. A number of such cases have been published as observational reports and also appear in diagnostic laboratory surveys and reports. A fine example is the diagnosis of Inherited Congenital Myoclonus (formerly known as Hereditary Neuraxial Oedema) in a neonatal Poll Hereford calf that presented as stillbirth in an experimental breeding herd for this disease, but the calf had also been infected by AKAV infection that resulted in severe hydranencephaly [83]. At necropsy, the presence of bilateral fractured coxae was consistent with the diagnosis of Inherited Congenital Myoclonus causing severe adduction of the hindlimbs in utero, as invariably occurs in this disorder [83]. However, the presence of severe hydranencephaly due to AKAV infection that had ablated the cerebral cortex, indicated that an intact cerebrum that could become oedematous was unnecessary for the signs of Inherited Congenital Myoclonus to occur. The importance of this serendipitous case was that it indicates that the metabolic error in Inherited Congenital Myoclonus was distal to the brain (and not involving oedema of the neuraxis), with subsequent studies demonstrating that the myoclonic signs were attributable to an inherited deficiency of spinal glycine receptor mediated inhibitory feedback of lower motor neurones [84].

A list of similar examples is likely to be exhaustive and difficult to access as such cases do not necessarily appear in a literature search. However, some cases have been published as regular scientific articles and are summarized here to provide an overview of BCM syndromes found in calves exposed to teratogenic virus, but scientific evidence of a causal association is lacking in all cases.

*Perosomus elumbis* This congenital syndrome is characterised by lack of the lumbar, sacral and coccygeal spinal cord and the corresponding vertebrae. The caudal half of the calf consequently consists of a soft tissue sack enclosing the abdominal organs and with dysplastic arthrogrypotic hind limbs attached to a malformed pelvis [85]. A case of perosomus elumbis with concurrent BVDV infection has been reported [86]. The cause of perosomus elumbis is at present unknown.

*Cyclopia* The most obvious finding in cyclopia cases is the presence of a single large eye located in facial midline. This defect has been reported in a calf with congenital antibodies against BVDV [87]. Bovine cyclopism develops due to damage to the developing foetal forebrain during a narrow period of vulnerability on or about GD 14 [88]. The presence of congenital antibodies in this case showed the foetus was exposed to BVDV much later, i.e. later than around GD 90, where the foetus becomes immunocompetent [2], so BVDV was superimposed on an already existing malformation.

*Congenital bone fragility* Congenital bone fragility was reported in a calf with persistent BVDV infection. Unilateral fractures of the metaphyseal region of femur and tibia was observed, but histology revealed a generalized segmental disruption of trabecular modelling, including loss of trabecular bone [89]. Growth arrest lines were present in the bones; a lesion associated with foetal BVDV infection that indicates that an infected foetus may undergo several crises with growth arrest [24]. However, the bone fragility was probably unrelated to the BVDV infection.

### Congenital syndromes resembling VICMs

Bovine VICMs are most readily characterized by a rather uniform spectrum of lesions in the CNS such as hydranencephaly and cerebellar hypoplasia. However, neither these lesions, nor the associated localized or generalized arthrogryposis of the axial and appendicular skeleton seen in SBV and AKAV infected foetuses are pathognomonic. Malformations of the CNS and the musculoskeletal system are the most commonly recorded congenital defects in cattle [90] and in addition to teratogenic viruses, a wide range of other causes including genetic defects may cause such lesions. An up-dated overview of genetic disorders can be found at http://omia.angis.org.au/home/. However, it is important to remember that even though some teratogenic and genetic causes of BCMs are known, a substantial number of at present unknown aetiologies most likely exists and remain to be diagnosed. Practitioners and diagnosticians require awareness of this dilemma as it may lead to misdiagnosis of the cause of BCMs and in case of inherited syndromes, lead to an unrecognised spread of the defect within a herd or breed. Expert advice should be sought and calves submitted for extended diagnostic investigation where surveillance programs for genetic diseases are in place.

A number of inherited congenital syndromes that closely resemble SBV-, AKAV- and AV-induced generalized arthrogryposis and distortion of the skeleton have been reported. The molecular background of these syndromes differs, but disturbed neuro-muscular development appears central in the pathogenesis. Inherited syndromes of tetramelic arthrogryposis and palatoschisis have been reported, especially in the Charolais and Hereford breeds e.g. [91–93], although similar non-specific syndromes may occur in most breeds [94]. Palatoschisis is not reported to be a part of VICMs.

A specific inherited defect belonging to the “arthrogryposis multiplex congenita syndrome” complex, originally termed “Curly calf syndrome”, has been reported in Angus cattle [95]. Affected stillborn fullterm calves have reduced birth weights (15–25 kg). The forelimbs are normally observed in fixed flexion, with hind limbs usually found fixed in extension. Lateral deviation of the facial bones is commonly observed, with severe rotational deviation of the cervical, thoracic and lumbar vertebral column and deformation of the ribcage and sternum secondary to the scoliosis, frequently observed. Mild hydrocephalus and palatoschisis may also be occasionally observed. This defect was transmitted by the elite US sire GAR Precision 1680 mainly through his son CA Future Direction 5321. The causal mutation has been identified, but not published (see Additional file 2).

Arachnomelia, an inherited syndrome recognized in the Brown Swiss and Simmental breeds, share some similarities with VICMs, such as kyphosis, scoliosis and tetramelic arthrogryposis. However, the long bones of the limbs are longer and thinner than normal and the maxillae and frontal bones are deformed [96, 97]; lesions that are usually not present in bovine VICM cases.

Genetic disorders resembling BVDV- and BTV-induced CNS lesions, identified as malformations mainly restricted to the CNS without associated arthrogryposis, do occur, although few have been reported. The worldwide occurrence of BVDV and its well-established association with cerebellar hypoplasia has probably out-numbered the sporadic cases of non-infectious aetiology, so cases of cerebellar hypoplasia are generally considered to be a VICM by practitioners and diagnosticians in regions where this infection is endemic. Cases of cerebellar hypoplasia in Hereford [98] and Shorthorn calves [95] and cerebellar abiotrophy in Angus cattle [99] occurring in a familial pattern that indicated a genetic aetiology rather than a teratogen, have been reported, although it is very likely that similar syndromes have occurred in many breeds.

Neuropathic hydrocephalus is an autosomal recessive defect in Angus cattle that is characterised by foetal growth retardation and an extremely severe hydrocephalus with doming of the calvarium. The malformed skull causes dystocia and drainage of the cerebrospinal fluid is required to achieve vaginal delivery [95]. The causal mutation of this defect is known, but not published (see Additional file 3).

Other likely genetic defects characterised by hydrocephalus such as two distinct syndromes in Hereford cattle have been reported in older literature [100]. However, since hydrocephalus and hydranencephaly are often considered to be caused by viral teratogens detailed investigation on sporadic cases is rarely reported as the aetiology mostly remains unsolved. However importantly, the eventual recognition of inherited neuropathic hydrocephalus in Angus cattle despite high foetal mortality rates, clearly demonstrates that inherited forms can emerge rapidly and that it is likely that other entities remain to be recognized.

### How to approach suspected cases of VICM in cattle

Arthrogrypotic syndromes are usually readily recognized by clinicians but can be difficult to differentiate aetiologically. However, malformations that only involve the CNS are more challenging to diagnose in large animal practice, especially if the shape of the calvarium is normal. Hydrocephalus, hydranencephaly, porencephaly, and cerebellar hypoplasia may be the only gross lesions present in bovine VICMs, especially in BVDV-, BTV- and AKAV- exposed foetuses. Clinical signs vary depending on the extent of the lesions, but are usually non-specific and affected calves may live for several months. Appropriate diagnostics requires examination of the brain and although the skull can be opened in the field, detailed examination usually requires submission to a laboratory. Oozing of larger volumes of clear fluid through the *foramen magnum* during decapitation or a domed calvarium often indicates the presence of hydrocephalus or hydranencephaly. Palatoschisis has not been reported in association with VICMs. Ocular lesions such as retinal dysplasia and retinitis in combination with brain malformation and/or encephalitis are regarded as highly indicative for foetal BVDV exposure. However, the diagnosis of VICMs in cattle based on only gross lesions is insufficient even in cases with arthrogryposis and is not recommended unless the malformed calves have characteristic lesions *and* occur during a well-defined outbreak, preferably supported by serological or virological evidence of recent infection.

Veterinary practitioners should seek advice from experts in bovine teratology when encountering BCMs. Submission of materials should ideally consist of the entire calf, maternal blood samples and anamnesis, including herd status for viral diseases, vaccination and pedigree data. Euthanasia by the use of a captive bolt pistol should be avoided and submission of fresh, if possible cooled, rather than frozen cadavers is preferred. If submission of the carcass isn’t feasible, photos and description of gross lesions and submission of the head, lung, spleen and precolostral blood sample/pleural effusion sample may be used to confirm VICMs, but access to the entire carcass is needed to perform a thorough investigation.

The geographical region may be of some use for the practitioner to rule out certain VICMs. BVDV has been eradicated in some countries and the occurrence of the vectors of SBV, BTV, AKAV and AV may be restricted to certain regions or habitats, although the recent transmission of BTV and SBV in Europe indicates that such preconceived notions can be challenged.

The season at which malformed calves are delivered may also indicate if an insect vector transmitted virus such as SBV, BTV or AKAV may be implicated. Fullterm malformed offspring may be delivered in a wide time-frame or “window”, from around 80–220 days after foetal exposure, so the vectors must have been active at that time of gestation. Although the risk of transmission is higher during periods with high environmental temperatures, transmission may occur even during periods with temperatures of 5–9 °C [101], which is not unusual in many regions of Europe during the winter period. The season of birth of defective offspring can therefore not be used to rule out a VICM in cattle although the risk is higher in some periods. Further, in AKAV infection in herds with extended mating seasons, the delivery of malformed calves may indicate that calves in mid-gestation were infected and the virus has induced lesions only in the spinal cord (between GDs 103 and 173), whereas calves conceived later in the breeding season may have been exposed earlier in the development of their CNS, so lesions will only involve the cerebrum and calves will likely present within a few weeks with hydranencephaly (following infection between GDs 79 and 104).

The presence of maternal antibodies may indicate a specific viral aetiology in regions where the infection is not enzootic and where vaccination hasn’t been performed. As foetal lesions develop several months before parturition, sufficient time exists from the onset of infection to delivery of malformed offspring, for virus-specific antibodies to be produced. Congenital antibodies in either precolostral serum or pleural/pericardial effusion sampled during necropsy are consistent with intrauterine infection of the foetus after it has become immunocompetent, i.e. around GD 80–90 [2, 102]. However, in VICMs occurring prior to the onset of foetal immune competency such as BVDV infection, the virus may escape recognition by the foetal immune system, leading to persistently infected, but seronegative individuals (so-called “persistently infected calves”). Infected foetuses may also be aborted before a significant antibody response has developed. In addition to serology, it is therefore imperative that examination for virus or viral antigen is performed, e.g. by PCR, cell culture or in situ detection methods. However, as previously advised, it is important to stress that identification of the presence of infection, either as virus or antibodies, doesn’t necessarily imply that the virus is the cause of the malformation.

Examination of pedigrees for inbreeding is sometimes used as a practical tool to differentiate between inherited and non-inherited syndromes, including VICMs. However, again, this may be unreliable as although inbreeding may indicate the possibility of a genetic aetiology, it does not preclude other causes of the BCM. Furthermore, a number of described BCMs of genetic aetiology have occurred in large numbers without readily identifiable inbreeding having occurred, such as a common ancestor being present within 2–3 generations [96].

## Conclusions

VICMs caused by BVDV, SBV, BTV, AKAB and AV mainly involve the CNS as the developing brain is generally vulnerable to the teratogenic properties of these viruses from around GD 60–180 but with individual viral time preferences. These viruses may cause necrosis of the neuroparenchyma that may be exacerbated by the foetal inflammatory response, leading to disturbance of tissue integrity, grossly recognized as hydranencephaly, hydrocephalus (*ex vacuo*), porencephaly, and cerebellar hypoplasia, although the latter lesion is rarely observed in AKAV infection. Recognition of these lesions often requires inspection of the brain, but may in some cases be associated with a domed calvarium, usually indicating the presence of hydrocephalus. As SBV, AKAB and AV also causes damage to the spinal cord leading to disturbed innervation of the skeletal musculature, these VICMs may be recognized clinically as arthrogryposis of the limbs and deviation of the vertebral column, such as torticollis and scoliosis.

None of the gross lesions are pathognomonic for VICMs as a number of genetic disorders resemble the congenital teratogenic syndromes. It is therefore recommended to seek advice from experts in bovine teratology and genetic diseases before diagnosing VICMs in practice and if possible, submit the animal for laboratory examination. As the association of a suspected VICM as the cause of a BCM is best confirmed by demonstration of lesions known to be associated with viral infection, supported by demonstrating the presence of congenital antibodies or virus indicative of foetal infection, the diagnosis of a congenital syndrome should be done carefully and be evidence based.

## Additional files

10.1186/s13028-015-0145-8 The ‘brevitarsis line’ displaying the northern distribution of the *Culicoides* sp. midges responsible for infections of ruminants with Akabane virus in Australia.
10.1186/s13028-015-0145-8 Fact sheet on arthrogryposis multiplex from the American Angus Association. Downloaded from http://www.angus.org/pub/AM/AMFactSheet.pdf, 8 May 2015.
10.1186/s13028-015-0145-8 Fact sheet on neuropathic hydrocephalus from the American Angus Association. Downloaded from http://www.angus.org/pub/nh/nhfactsheet.pdf, 8 May 2015.

## Abbreviations

AKAV: Akabane virus
AV: Aino virus
BCM: bovine congenital malformation
BT: bluetongue
BTV: blue tongue virus
BTV-8: blue tongue virus serotype 8
BVDV: bovine virus diarrhea virus
CNS: central nervous system
Cp: cytopaphogenic
GD: gestation day
NAMP: National Arbovirus Monitoring Program
Ncp: non-cytopathogenic
NSW: New South Wales, Australia
SBV: Schmallenberg virus
PCR: polymerase chain reaction
VICM: virus-induced congenital malformation
