# Supplementary material for: Virus-induced congenital malformations in cattle
Source: Acta Vet Scand. 2015 Sep 24;57(1):54. doi: 10.1186/s13028-015-0145-8 (PMC4581091; doi:10.1186/s13028-015-0145-8)
Supplement: Supplementary file 1 — 10.1186/s13028-015-0145-8 The ‘brevitarsis line’ displaying the northern distribution of the Culicoides sp. midges responsible for infections of ruminants with Akabane virus in Australia. [file 13028_2015_145_MOESM1_ESM.pdf]

Additional file 1. Distribution of Akabane virus north of the so-called ‘brevitarsis line’ in Australia in 2013-14 and location of Hunter Valley (arrow).

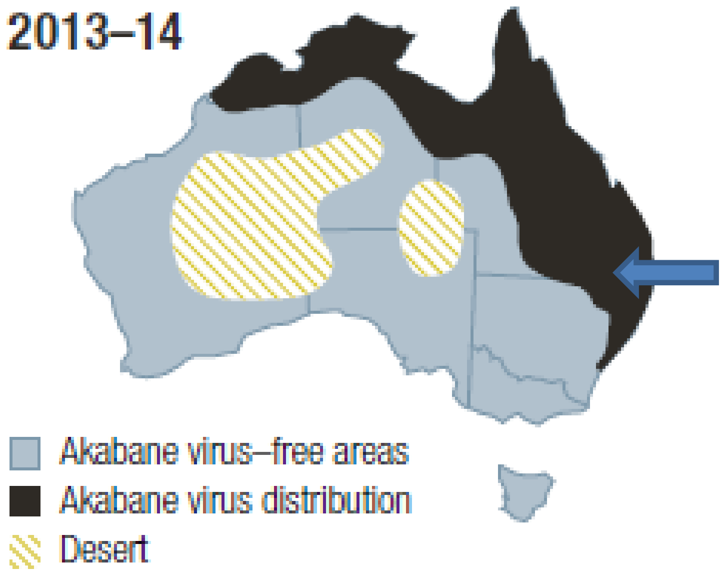

From NAMP 2013-14 Report, Animal Health Australia.
http://www.animalhealthaustralia.com.au/wp-content/uploads/2015/01/FINAL_web_namp1314_4.pdf
