# Supplementary material for: Virus-induced congenital malformations in cattle
Source: Acta Vet Scand. 2015 Sep 24;57(1):54. doi: 10.1186/s13028-015-0145-8 (PMC4581091; doi:10.1186/s13028-015-0145-8)
Supplement: Supplementary file 2 — 10.1186/s13028-015-0145-8 Fact sheet on arthrogryposis multiplex from the American Angus Association. Downloaded from http://www.angus.org/pub/AM/AMFactSheet.pdf, 8 May 2015. [file 13028_2015_145_MOESM2_ESM.pdf]

American Angus Association®
Arthrogryposis Multiplex (AM)
Fact Sheet

The following fact sheet was developed to respond to questions commonly asked by American Angus Association members. Additional information may be found online at www.angus.org.

What is Arthrogryposis Multiplex (AM)?
AM was recognized as a genetic defect on September 16, 2008. Calves are born dead or die shortly after birth. The spine and legs appear crooked or twisted and the joints of the legs are often fixed in position. Front legs are contracted and rear limbs may be contracted or extended. Calves are small and appear thin due to limited muscle development. There may be a cleft affecting the nose or palate.

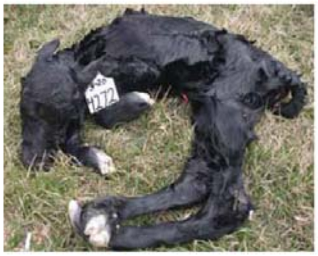
Figure 1

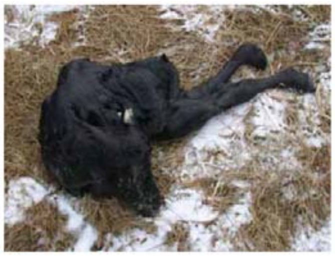
Figure 2

What causes AM?
AM is caused by a recessive mutation on a single cattle chromosome. Cattle that are homozygous for the mutated gene will exhibit AM.

What is an AM carrier?
For the purpose of this response, an AM carrier is an Angus or Angus-cross cow, heifer, bull or steer that carries the recessive AM mutation in their DNA.

Why are carriers of AM important?
Carriers of AM used in breeding programs (registered or commercial) are responsible for propagating the recessive mutation within the cattle population.

What does an AM carrier look like?
An AM carrier looks perfectly normal; there is nothing in the way an animal looks (its phenotype) that indicates that the animal is a carrier of the AM mutation.

If a cow has an AM calf, what does that mean?
If a cow has an AM calf, and if it is the cow's natural calf, it means that the cow is a carrier of the AM mutation and the sire of the calf is also an AM carrier.

If a recipient cow has an AM calf, what does that mean?
If a recipient cow has an AM calf, it means only that both the donor cow and the sire of the calf are carriers of the AM mutation. It doesn't tell you anything about the AM carrier status of the recipient cow.

If a bull sires an AM calf, what does that mean?
If a bull sires an AM calf, it means that the bull is a carrier of the AM mutation and that the dam of the calf is also an AM carrier.

I have never had an AM calf. Does that mean my cows are non-carriers?
Not necessarily.

What is the risk of having an AM calf if I breed an AM carrier cow to an AM carrier bull?
Every time you breed a carrier to a carrier, there is:
- A 25% risk of having a dead AM calf;
- A 50% risk of having an otherwise normal-looking calf that carries the AM mutation;
- A 25% chance that you will have a non-carrier calf.

If I breed an AM carrier cow to an AM carrier bull and have three live calves, will the fourth calf have AM?
The risk is the same every time you breed a carrier to a carrier. There is always a 25% risk of having a dead AM calf, a 50% risk of having a carrier calf, and a 25% chance of having a non-carrier calf.

If I breed an AM carrier cow to a non-carrier bull, what is the chance of having an AM calf?
Zero. You will never have an AM calf if you breed a carrier cow to a non-carrier bull. (excluding the possibility of a spontaneous mutation)

If I breed an AM carrier cow to a non-carrier bull, what is the risk of having a carrier calf?
Every time you breed a carrier cow to a non-carrier bull there is:
- A 50% risk of having a normal-looking calf that carries the AM mutation; and
- A 50% chance you will have a non-carrier calf.

Is there a test to identify AM carriers?
Yes. A DNA test is available to determine if an animal carries the AM mutation in their DNA. The type of DNA sample required to perform the test varies from lab to lab but includes; hair root samples, blood-spot or FTA cards, whole blood in "purple –top" tubes, tissue samples from ears and semen samples.

An article outlining how to collect DNA samples was published in the January 2010 Angus Journal. It can be found here.

What do I do with the confirmed non-carrier females in my herd?
If the females are tested non-carriers and they are bred to non-carrier bulls, they will never produce affected AM calves or carriers. These non-carrier females can be used throughout your breeding program with no risk of propagating the AM mutation.

What do I do with confirmed female carriers in my herd?
You have several options:
- If you have a cow that carries the AM mutation and you want to produce calves from her; you must make a commitment to test all offspring retained for breeding;
- If you have both a registered and a commercial herd, retain your carrier cows in the commercial herd, breed to a non-carrier bull, and test any calves retained for breeding purposes;
- If you always breed your carrier cows to a non-carrier bull, you will never have an AM calf. Then, treat the resulting calves as market animals, not as breeding stock.
- Use your AM carrier cows as ET recipients. As a recipient female, she has no genetic effect on the embryo calf she raises.

AM potential carrier report
AAA Login users can access an interactive tool to generate a report of owned animals and their Arthrogryposis Multiplex (AM) status based on the AM test results received to date. From the AAA Login menu, go to the "interactive" section and click on "Potential Carrier Report AM/NH" If you are not a current AAA Login user, you can sign up to create an online profile at *www.angusonline.org*

What is the AAA registration policy regarding AM?
AMC- Arthrogryposis Multiplex Carrier, has been tested and carries the AM mutation.
AMF- Arthrogryposis Multiplex Free, has been tested and does not carry the AM mutation.

		**One or both parents test AMC (confirmed carriers)**
**Heifers**		If born on or before 12/31/2011, must be tested and can be registered regardless of the test outcome.
**Heifers**		If born on or after 1/1/2012, must be tested and only those that test AMF can be registered.
**Bulls**		If born on or before 12/31/2009, must be tested and can be registered regardless of the test outcome.
**Bulls**		If born on or after 1/1/2010, must be tested and only those that test AMF can be registered.
**E.T. Calves**		Registration is based on the date of birth and if they are sired by a sire that is an A.I. bull as described below.
**Steers**		No test required.
**Potential Carriers and "Pop Ups"**		Any animal that traces to a confirmed (tested) animal will be classified as a "Potential Carrier" unless an intervening ancestor has tested "Free" of AM. Beginning 7/1/2009, a "pop up" notation will appear on the registration certificate, performance pedigree and electronically on the web site pedigree. Ancestral based potential carriers (vs. a potential carrier due to the fact that one or both parents are confirmed carriers) are not required to do testing on their progeny but are encouraged to test in the notation to confirm the absence or presence of AM.
**A.I. Sire that are confirmed carriers**		Calves cannot be registered that are conceived more than 60 days after the date a non-owned bull (a bull that would require an A.I. Service Certificate) is listed as a carrier animal (AMC).

The following labs are authorized for AM

MMI Genomics
1756 Picasso Avenue
Davis, CA 95618
(800) 311-8808 ext 3016
http://www.mmigenomics.com/AM.html

AgriGenomics
2399 N. 1000 E. Rd.
Mansfield, IL 61854
217-762-9808
http://www.agrigenomicsinc.com

Pfizer Animal Genetics
333 Portage Road
Kalamazoo, MI 49007-4931
1-877-BEEF DNA
1-877-233-3362
Fax: 269-833-1197
http://www.pfizeranimalgenetics.com

IGENITY
4701 Innovation Drive, CB 101
Lincoln, NE 68521
1-877-IGENITY
1-877-443-6489
http://www.igenity.com

GeneSeek
4665 Innovation Dr. Suite 120
Lincoln NE 68521
402-435-0665
www.geneseek.com

The following groups collaborate with GeneSeek, Inc., to collect and provide samples for AM testing:
- Stockman's Resource Center LLC
  2371 330th Street
  Eddyville, Iowa 52553
- stockmansresource@hotmail.com
  www.stockmansresource.com
  Office phone: 641-969-4111
  Mobile: 641-660-0771
- SEK Genetics
  Don Coover
  9525 70th Rd.
  Galesburg, KS 66740
  don@sekgenetics.com
  Phone: 800-443-6389
- Genex Cooperative, Inc.
  Headquarters:
  100 MBC Drive
  Shawano, WI 54166
  Phone: 888-333-1783
  Fax: 715-526-3219
  info@crinet.com
