# Supplementary material for: Virus-induced congenital malformations in cattle
Source: Acta Vet Scand. 2015 Sep 24;57(1):54. doi: 10.1186/s13028-015-0145-8 (PMC4581091; doi:10.1186/s13028-015-0145-8)
Supplement: Supplementary file 3 — 10.1186/s13028-015-0145-8 Fact sheet on neuropathic hydrocephalus from the American Angus Association. Downloaded from http://www.angus.org/pub/nh/nhfactsheet.pdf, 8 May 2015. [file 13028_2015_145_MOESM3_ESM.pdf]

American Angus Association®
Neuropathic hydrocephalus (NH)
Fact Sheet

ANGUS
THE BUSINESS BREED

The following fact sheet was developed to respond to questions commonly asked by American Angus Association members. Additional information may be found online at www.angus.org.

What is Neuropathic Hydrocephalus (NH)?
NH was recognized as a genetic condition on June 12, 2009. Calves that are carried are born near term and may have 25-35 pound birth weights. Some evidence also points toward possible early abortions due to the defect. The cranium is markedly enlarged (volleyball to basketball sized). The bones of the skull are malformed and appear as loosely organized bony plates that fall apart when the cavity is opened. The cranial cavity is filled with fluid and no recognizable brain tissue is evident. The spinal canal is also dilated and no observable spinal tissue is found.

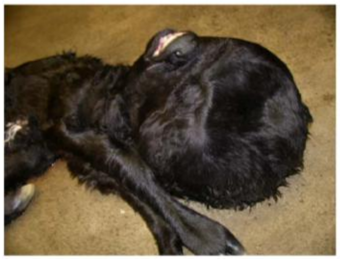
Figure 1

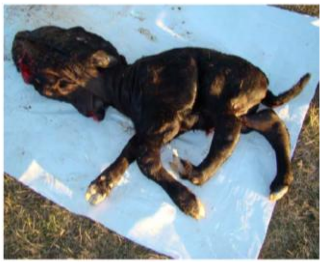
Figure 2

What causes NH?
NH is caused by a recessive mutation on a single cattle chromosome. Cattle that are homozygous for the mutated gene will exhibit NH.

What is an NH carrier?
For the purpose of this response, an NH carrier is an Angus or Angus-cross cow, heifer, bull or steer that carries the recessive NH mutation in their DNA.

Why are carriers of NH important?
Carriers of NH used in breeding programs (registered or commercial) are responsible for propagating the recessive mutation within the cattle population.

What does an NH carrier look like?
An NH carrier looks perfectly normal; there is nothing in the way an animal looks (its phenotype) that indicates that the animal is a carrier of the NH mutation.

If a cow has an NH calf, what does that mean?
If a cow has an NH calf, and if it is the cow's natural calf, it means that the cow is a carrier of the NH mutation and the sire of the calf is also an NH carrier.

If a recipient cow has an NH calf, what does that mean?
If a recipient cow has an NH calf, it means only that both the donor cow and the sire of the calf are carriers of the NH mutation. It doesn't tell you anything about the NH carrier status of the recipient cow.

If a bull sires an NH calf, what does that mean?
If a bull sires an NH calf, it means that the bull is a carrier of the NH mutation and that the dam of the calf is also an NH carrier.

I have never had an NH calf. Does that mean my cows are non-carriers?
Not necessarily.

What is the risk of having an NH calf if I breed an NH carrier cow to an NH carrier bull?
Every time you breed a carrier to a carrier, there is:
- A 25% risk of having a dead NH calf;
- A 50% risk of having an otherwise normal-appearing calf that carries the NH mutation;
- A 25% chance that you will have a normal-appearing, non-carrier calf.

If I breed an NH carrier cow to an NH carrier bull and have three live calves, will the fourth calf have NH?
The risk is the same every time you breed a carrier to a carrier. There is always a 25% risk of having a dead NH calf, a 50% risk of having a carrier calf, and a 25% chance of having a non-carrier calf.

If I breed an NH carrier cow to a non-carrier bull, what is the chance of having an NH calf?
Zero. You will never have an NH calf if you breed a carrier cow to a non-carrier bull. (excluding the possibility of a spontaneous mutation)

If I breed an NH carrier cow to a non-carrier bull, what is the risk of having a carrier calf?
Every time you breed a carrier cow to a non-carrier bull there is:
- A 50% risk of having a normal-appearing calf that carries the NH mutation; and
- A 50% chance you will have a non-carrier calf.

Is there a test to identify NH carriers?
Yes. A DNA test is available to determine if an animal carries the NH mutation in their DNA. The type of DNA sample required to perform the test varies from lab to lab but includes; hair root samples, blood-spot or FTA cards, whole blood in "purple –top" tubes, tissue samples from ears and semen samples.

A video on ***www.angus.org*** explaining how to collect the sample can be found **here**.

What do I do with the confirmed non-carrier females in my herd?
If the females are tested non-carriers and they are bred to non-carrier bulls, they will never produce affected NH calves or carriers. These non-carrier females can be used throughout your breeding program with no risk of propagating the NH mutation.

What do I do with confirmed female carriers in my herd?
You have several options:

- If you have a cow that carries the NH mutation and you want to produce calves from her; you must make a commitment to test all offspring retained for breeding; (check policy regarding registration requirements)
- If you have both a registered and a commercial herd, retain your carrier cows in the commercial herd, breed to a non-carrier bull, and test any calves retained for breeding purposes;
- If you always breed your carrier cows to a non-carrier bull, you will never have an NH calf.
- Use your NH carrier cows as ET recipients. As a recipient female, she has no genetic effect on the embryo calf she raises.

NH potential carrier report & potential carrier management tool

AAA Login users can access interactive tools to generate a report of owned animals and their Neuropathic Hydrocephalus (NH) status based on the NH test results received to date. From the AAA Login menu, go to the "interactive" section and click on "Potential Carrier Report AM/NH/CA/DD/M1/D2" or "Potential Carrier Management Tool (PCMT)." The PCMT can identify those animals in your herd that have the most descendants in your herd and would be the most logical animal to start a testing scheme should you decide to test for a particular genetic condition. If you are not a current AAA Login user, you can sign up to create an online profile at *www.angusonline.org.*

What is the AAA registration policy regarding NH?

		**If a calf is a potential carrier submitted for registration after 9-13-12.**
**Heifers**		Must be tested and can be registered regardless of the test outcome.
**Bulls**		Must be tested and only those that test NHF can be registered.
**E.T. Calves**		Registration is based on sex of calf and if they are sired by a bull that is an A.I. sire as described below.
**Steers**		No test required.
**A.I. Sires that are confirmed carriers**		Calves cannot be registered that are conceived more than 60 days after the date a non-owned bull (a bull that would require an A.I. Service Certificate) is listed as a carrier animal (NHC).
**Definitions**		NHC - NH Carrier, has been tested and carries the NH mutation.<br>NHF - NH Free, has been tested and does not carry the NH mutation.<br>NHP - NH Potential Carrier, animal that traces to one or more confirmed tested carrier animals in its pedigree that have no intervening ancestors that have been tested free of NH.

Two Testing Options

1. Submit Samples through American Angus Association/AGI

Use AAA Login to order tests. Samples are submitted to the American Angus Association and archived for future testing requests. Login at *www.angusonline.org* and use menu option: Order --Testing for AM/NH/CA/DD/M1/D2.

2. Additional Authorized Labs for Neuropathic Hydrocephalus (NH) Testing

Below are the labs currently authorized for NH testing by the American Angus Association. Consult the respective lab web sites for information on DNA preferred sample types, sample submission forms, pricing information and complete instructions on how and where to submit samples for testing. In choosing a lab, members of the Association are urged to read and carefully consider any language on a given lab's submission form (for the NH test) or on its accompanying "Terms and Conditions" that relates to any lab's alternative use of the DNA samples being submitted.

The following labs are authorized for NH

AgriGenomics
2399 N. 1000 East Rd.
Mansfield, IL 61854
217-762-9808
Fax: 217-762-9809
http://www.agrigenomics-inc.com

GeneSeek
4665 Innovation Dr. Suite 120
Lincoln NE 68521
402-435-0665
Fax: 402-435-0664
www.geneseek.com

Zoetis
333 Portage Road, Bdg. 300
Kalamazoo, MI 49007-9970
877-233-3362
Fax: 269-833-4711
http://www.zoetis.com
